# Indigenous-Amazonian Traditional Medicine’s Usage of the Tobacco Plant: A Transdisciplinary Ethnopsychological Mixed-Methods Case Study

**DOI:** 10.3390/plants12020346

**Published:** 2023-01-11

**Authors:** Ilana Berlowitz, Ernesto García Torres, Caroline Maake, Ursula Wolf, Chantal Martin-Soelch

**Affiliations:** 1 Faculty of Medicine, University of Zurich, 8057 Zurich, Switzerland; 2 Department of Biomedical Research, University Hospital Bern, 3010 Bern, Switzerland; 3 Traditional Amazonian Healer, Loreto, Iquitos 1600, Peru; 4 Institute of Complementary and Integrative Medicine, University of Bern, 3012 Bern, Switzerland; 5 Department of Psychology, University of Fribourg, 1700 Fribourg, Switzerland

**Keywords:** tobacco, *Nicotiana*, *N. rustica*, mapacho, indigenous medicine, traditional medicine, Amazonian medicine, Peru, Amazon, ayahuasca, psychoactive, case study, mixed-method, experience-sampling, ecological momentary assessment

## Abstract

Harmful usage of tobacco is a global public health problem associated with adverse health effects and addiction. Yet, in the Peruvian Amazon, the native region of *Nicotiana rustica* L., this plant is used in remarkably different manners: it is considered a potent medicinal plant, applied in liquid form for oral ingestion to treat mental health problems, a common and ancient healing practice in this region. Using a transdisciplinary field research approach with mixed ethnopsychological methods, this work aimed to report for the first time a case study in this context. The intervention took place in the Peruvian Amazon (Loreto) and involved ritual tobacco ingestion in a weeklong retreat-like frame, administered by a specialized traditional Amazonian healer. The patient was a 37-year-old woman with diagnosed mood, anxiety, and attention deficit disorders, as well as a chronic somatic condition. We applied qualitative experience-sampling during and quantitative symptom assessments pre- and post-treatment. Our findings offer a detailed description of the experiential therapeutic process during the treatment week and suggest clinically relevant improvements in patient well-being. This work is significant in view of the globally prevalent harmful uses of tobacco and the current scientific trend of revisiting herbal psychoactives (e.g., cannabis, psilocybin) for their therapeutic potentials.

## 1. Introduction

Harmful usage of tobacco is associated with addiction, risk for non-communicable diseases, and, with over a billion smokers worldwide, is a public health problem of global concern [1,2,3,4,5]. Yet, in the Peruvian Amazon region, the hypothesized birthplace of *Nicotiana rustica* L. [6,7], this plant is associated with a remarkably different usage and repute: it is seen as an exceptional medicinal plant, described as a ‘Master Plant’ [8,9] and as the main curing tool of Amazonian healers [10]. The Huni Kuin consider tobacco the ‘quintessential healing substance’ [11] and the Amazonian Keshwa call it the ‘father of all plants’ [10,12]. Ethnographic accounts of tobacco as a sacred and medicinal plant are found across the Americas, in conjunction with millennia-old traditions [7,13]. However, it is particularly in Amazonian traditional healing that tobacco is fundamental; so fundamental that in various Indigenous languages the generic term for ‘healer’ is etymologically linked to the word for ‘tobacco’, for instance in the Yuracaré language, where *korrë-n-chata* (‘healer’) literally translates as ‘he who eats tobacco’; or in the Asháninka and Matsigenga languages, where the term for healer (*sheripiari* and *seripigari*, respectively) translates as ‘the one intoxicated by tobacco’ [8,14,15,16,17]. The oldest archeological evidence of human tobacco use dates to 12,300 years ago [18,19,20]. The Iberian colonizers encountered the plant in the 15th century, as it was used in healing and cultural practices of the American Natives, and brought it to Europe, from where it quickly spread to the rest of the world [6,21]. In spite of an initial interest in its medicinal value [22], the plant’s large-scale commodification, and its global marketing by an increasingly transnational tobacco industry [23,24,25,26,27,28], propagated hedonic uses; uses which, in direct contradistinction to the plant’s origins in Indigenous medicine, later turned out to be detrimental to health [8,22]. The loss of Indigenous medicine knowledge on how to prepare and apply the plant for therapeutic purposes may have been crucial in what transformed tobacco from local medicinal plant to global substance of abuse.

Traditional or complementary medicines play an increasingly important role in global healthcare [29,30,31,32], with the World Health Organization (WHO) regarding their inclusion critical for universal health coverage [33]. This is particularly relevant also in the mental health domain, where treatments are known to be underprovided globally (cf. worldwide treatment gap in mental health [34,35,36,37,38,39]). In parallel, in the context of what is often labeled a ‘psychedelic renaissance’, compounds acting on the nervous system, which have formerly been known only for their harmful uses (e.g., psilocybin, cannabis; but also non-botanical compounds such as 3,4-methylenedioxymethamphetamine [MDMA] or lysergic acid diethylamide [LSD]), are now investigated for their therapeutic benefits in rapidly growing numbers of clinical trials [40,41,42,43,44,45,46,47,48,49,50,51,52,53,54,55]. In this context Amazonian traditional medicine, due to ayahuasca, has recently become the object of much scientific attention. Made of *Banisteriopsis caapi* and admixture plants (such as *N*,*N*-Dimethyltryptamine [DMT]-containing *Psychotria viridis*) [56,57,58,59,60], ayahuasca is a psychoactive brew invented by and used in Amazonian Indigenous medicine—the same medicine system as tobacco. Increasing clinical research points to benefits for depressed patients, anxiety, or addictive disorders [50,51,52,53,54,55,61,62,63,64,65,66,67,68,69], while concomitantly, growing numbers of international health-seekers each year travel to the Amazon to look for healing with Amazonian medicine means [66,70,71,72,73,74,75], which, given the plant’s central position in this medicine, often includes tobacco-based treatment. However, in contrast to the bourgeoning research on ayahuasca-based therapies, Amazonian treatments involving tobacco have so far been scientifically underexplored.

The current work aimed to report for the first time a case study in this context. In a previous paper [76] we provided an overview of clinically relevant aspects of Amazonian tobacco uses, including indications, contraindications, effects, risks, and adverse effect, in conjunction with different forms (solid, liquid, smoke) and routes of administration (topical, oral, intranasal, etc.). Among these applications, for the mental health domain particularly orally administered liquid-form tobacco (subsequently: “drinking tobacco”) was emphasized. The present work thus focused on this application. We conducted a case study in transdisciplinary collaboration [77,78,79] with a so-called *Maestro Tabaquero*, a traditional Amazonian healer whose medical specialization focuses on tobacco (a common name for *N. rustica* in this Amazonian region is *mapacho*; other names reported in the literature from various Indigenous American groups include for instance *sacha tabaco, yé, ya, tsalagayun’li, semaa, asemaa, real tobacco*, *Native tobacco, wild tobacco, Aztec tobacco, Hopi tobacco* [7,9,10,80,81,82,83,84,85,86,87]. Using a mixed-methods field research approach, we aimed to describe the therapeutic process experience and symptom changes of a patient receiving a weeklong treatment with oral tobacco by a Peruvian-Amazonian *Maestro Tabaquero*.

## 2. Results

### 2.1. Treatment Description and Setting

The treatment took place at the facilities of the *Maestro Tabaqueo* in the rural Maynas region (Loreto Province) of the Peruvian Amazon. For the eight days of the retreat-like intervention, the patient was accommodated in a simple wooden hut where she remained in solitude, as required by the traditional therapeutic protocol locally known as *dieta* (Spanish for ‘diet’). As the name suggests, the Amazonian *dieta* entails a strict nutritional regime for the time of the treatment (only plain vegetable foods; no salt, sugar, spices, oil, etc.), as well as the maintenance of certain rules (e.g., prohibitions on certain foods, sexual abstinence) for some weeks after the treatment (for further detail on Amazonian dietary practices in conjunction with psychoactive plant medicines, see [88,89]).

During a typical day of treatment, the patient spent the day in solitude in her hut; she was brought food twice a day (early morning and late afternoon), and each day was visited by the *Tabaquero* around midday. Table 1 shows brief descriptions of the healer’s daily visits to the patient based on the notes of an external observer who assisted the visits with Spanish–English translations. The *Tabaquero* would first inquire about the patient’s well-being and offer the opportunity to ask questions. He would then proceed to serve the tobacco remedy, an extract of cured tobacco leaves he had prepared at the outset of the treatment week. The dosage per serving was determined by the *Tabaquero* in the moment of his visits, based on his clinical observations at that time. The latter may be unlike standard biomedical or psychological criteria, involving heuristics from traditional Amazonian medicine (e.g., examination of patient’s subtle energy), which could be essential to the therapeutic benefit and safety of the tobacco-based treatment. The procedure for serving the tobacco was performed via traditional ritual techniques (see Table 1). Thereafter, the *Tabaquero* would encourage the patient to drink water, which was usually followed by emesis (plant-induced emesis is a common practice in traditional Amazonian medicine associated with depuration [90,91]). On the eighth day of the treatment, the *Tabaquero* performed a brief ritual procedure that marked the end of the retreat (*corte*, ‘cutting’ the diet), after which the patient was served a normal meal (with salt).

### 2.2. Participant

The participant (subsequently: T.P. or ‘the patient’) was a 37-year-old woman who, at the time of the study was single and living by herself. She was a licensed nurse with substantial work experience in a specialized field of nursing (not mentioned here to guarantee anonymity) but described herself as a ‘nurse in transition’, as she recently lost a job and at the time of the study was unemployed. *Clinical history.* In her youth, T.P. had received diagnoses of dysthymia, anxiety disorder (NOS), and attention deficit/hyperactivity disorder (ADHD). In terms of somatic health, she had been diagnosed with hypermobile Ehlers–Danlos syndrome (EDS, hEDS), a genetically based connective tissue disorder that is associated with a range of symptoms, including orthostatic intolerance, pain, spinal instability, dizziness, foggy head, and sleep disruption. Symptoms have been present since childhood but were recognized and diagnosed as EDS only a few years prior to the study. Her scores on diagnostic tests assessing current somatic and psychological symptomatology are reported in the following section. *Relevant prior treatments.* For EDS, there is no specific treatment available, but her doctor recommended T.P. take large amounts of water with electrolytes on a daily basis. T.P. considered this to help, explaining that she would otherwise often be bed-bound. For the mental health symptoms, T.P. had been prescribed antidepressants from age 18–35, the success of which she considered limited. She therefore had tried alternative treatments, including ketamine-assisted therapy in medical context as well as ceremonial ayahuasca in her home country, both of which she described as helpful but not entirely in terms of symptom remission. She had not been to the Amazon before and had never received any kind of tobacco-based treatment. She was not taking psychopharmaceuticals or other medications at the time of the study. *Primary treatment motive.* T.P.’s treatment motives were mainly psychological in nature. She mentioned depressed mood, ‘parts of myself that need healing’, dissatisfaction with familial and romantic relationships, as well as a lack of directionality about professional goals and sense of not knowing her place within her professional field.

### 2.3. Pre-Treatment Diagnostic Scales

The patient’s psychological and somatic symptoms prior to treatment (past month) are found in Table 2. Most noteworthy are elevated depression symptoms on the *Symptom Assessment* (SA-45 [97,98]) and elevated stress levels on the *Perceived Stress Scale* (PSS-10 [99]), as well as somewhat elevated interpersonal sensitivity (i.e., discomfort in relation to others), paranoid ideation, and obsessive/compulsive symptoms (SA-45). Self-reported somatic symptoms as per *Patient Health Questionnaire* (PHQ-15 [100]) were relatively low in severity (see Section 4 for description of all scales).

### 2.4. Comparison of Pre- and Post-Treatment Scores (Descriptivie)

**Figure 1:**
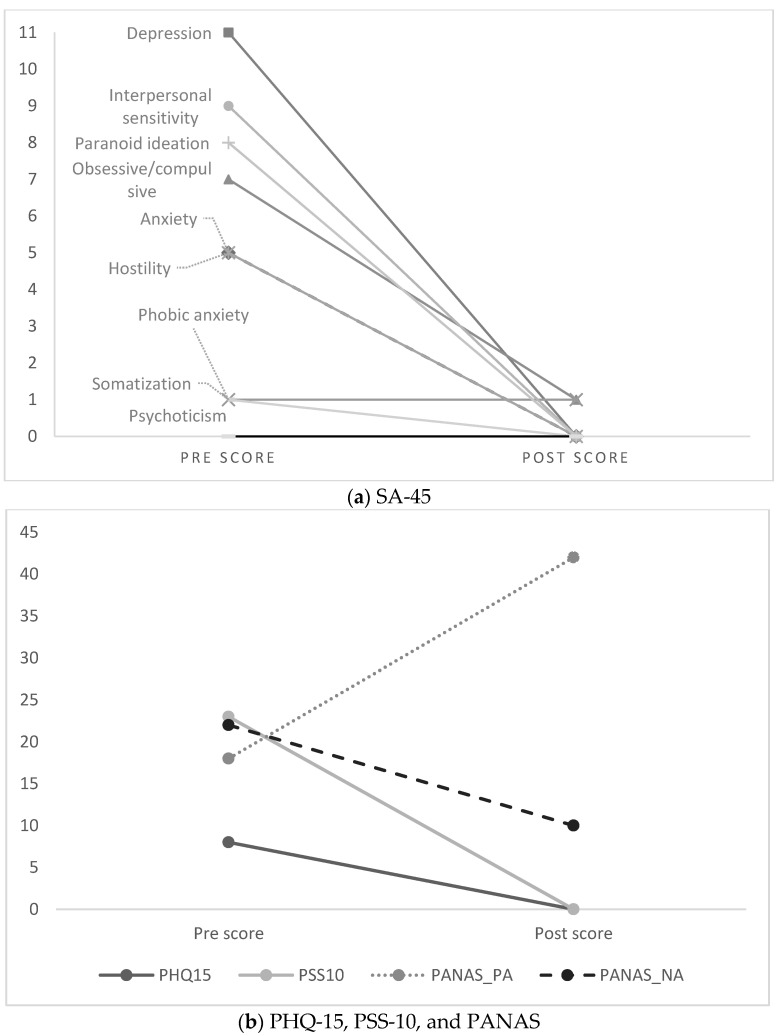
Pre- and post-treatment scores on psychological symptoms, somatic symptoms, stress, and affective states. Note: Pre- and post-treatment scores on (**a**) the 9 psychological symptom domains of the SA-45 = Symptom Assessment-45, and (**b**) the PHQ-15 = Patient Health Questionnaire-15, the PSS-10 = Perceived Stress Scale, and the PANAS = Positive And Negative Affect Schedule. Higher scores indicate higher severity of symptoms, except for PANAS positive affect.

Table 2 also shows psychological and somatic symptoms one day after treatment. The descriptive comparison points to substantial improvements at post- compared to pre-treatment, with the SA-45 Global Severity Index (an overall psychological symptom score) having diminished from 47 to 2. Figure 1a visualizes the changes on the nine symptom domains of the SA-45: Depressive symptoms, the highest domain at pre-treatment, showed a large amelioration after treatment, as did interpersonal sensitivity, paranoid ideation, obsessive/compulsive symptoms, anxiety, and hostility. Phobic anxiety did not change after the treatment, but was quite low to begin with, and similarly for somatization. The patient reported no psychotic symptoms. Psychological stress (PSS-10) and somatic symptom severity (PHQ-15) decreased to zero after treatment (see Figure 1b); the negative affect score of the Positive And Negative Affect Schedule (PANAS) [101] showed a two-fold decrease at post-treatment and positive affect on the PANAS had increased.

### 2.5. Experience during the Treatment: Ecological Momentary Assessment

Table 3 shows the content-analytic results of the ecological momentary assessment (EMA) [102], also called experience-sampling [103] (see Section 4). The main headings (first column) reflect the content segments of the EMA organized chronologically from morning to evening, namely ‘experiences of the previous night’, ‘the healer’s visit’, ‘immediate effects after drinking tobacco’, ‘experiences of the afternoon/evening’, and ‘changes in physiological functions across the day’. The subheadings (second column) reflect the EMA questions (abbreviated form). The table content itself (cells) then reflects the responses by the patient.

### 2.6. Patient Perspective at Post-Treatment

T.P. described feeling “emotionally steady and present” after the diet, noting “a kind of sustained attention that is very unusual for me”. She explained that in the course of the week certain personal tendencies of which she had not been aware, have become discernible. She also described important inspirational ideas in conjunction with her professional life. In this context she emphasized a strong sequence of insight particularly on the last evening of the diet, which she understood as a culmination of themes she had been working on during prior days (“as if filling in a multi-dimensional puzzle, all pieces came together”). It brought clarity about her true professional goals, as well as concrete ways to implement them, with a sense of excitement about the new perspective. T.P. further noted that, even though this had not been the motive of her consultation with the *Tabaquero*, there was also notable improvement of her symptoms related to EDS: she noted that she could now perform certain body movements that previously had been restricted and painful. She explained that the improvement has been progressive over the course of the seven days, but that in this context a marked shift was notable after the ‘*singada’* (tobacco ingestion via nostrils on day 5): she described that for the subsequent day large amounts of mucus was clearing from her sinuses and then she felt significantly lighter and clearer, the foggy head and dizziness characteristic of EDS diminished, and her balance and orientation improved. T.P. summarized the tobacco diet as “a very powerful treatment”.

## 3. Discussion

In spite of tobacco’s central role in traditional Amazonian medicine, and its importance in Indigenous American cultures in general [10,14,16,87], the plant’s therapeutic applications and associated patient reports have remained scientifically underexplored. Moreover, while the last decade has seen remarkable renewed interest in the clinical potentials of psychoactive herbal substances, such as ayahuasca, cannabis, or psilocybin, tobacco’s potentials in this context have so far been neglected. The current work reported for the first time a case study in this context. We employed a transdisciplinary fieldwork approach that combined qualitative and quantitative ethnopsychological methods to describe the therapeutic process of a patient treated with tobacco by a *Maestro Tabaquero* (local designation of traditional healer specialized in therapeutic uses of *N. rustica* [9]). The treatment took place in the Peruvian Amazon (Loreto, Maynas) and involved daily tobacco ingestion during a weeklong traditional dietary retreat (*dieta* [88,89]). We applied qualitative experience-sampling (EMA) to document the therapeutic process during the week, and a quantitative comparison of symptom ratings using validated psychological scales before and after treatment. The participant was a 37-year-old woman with diagnoses of mood, anxiety, and attention deficit disorder, as well as a chronic connective tissue syndrome.

Clinically relevant improvements in well-being were evident on the quantitative scales (including indicators of depression, interpersonal sensitivity, paranoid ideation, obsessive-compulsive tendencies, anxiety, hostility, and stress) and the patient’s qualitative descriptions in the post treatment interview. Daily experience-sampling (EMA) during the week pointed to a gradually increasing sense of calm, mental clarity, and general fortification (e.g., “I am stronger daily; I do not feel sick”, “calm, clear-headed”, “my patience and self-compassion are growing”). The patient described the treatment as an embodied experience [104] (“full body presence”, “the healing felt very grounded and physical”) with affective (e.g., “relief, gratitude, grief, joy came zipping out of me”) and cognitive (e.g., “my thoughts were remarkably clear and complete”) aspects (altered states). Spontaneously arising insights were commonly reported (e.g., “I have written 10 pages of deeply emotional insights”, “insight coming one after another after dark”), with themes relating to self-understanding (e.g., “I acknowledged my tendency to run, to seek novelty, to fluctuate […] the first time I have had real insight about this”, “my eagerness to please and be validated by others”), shifts of perception (“about my abundance: switch the perspective from having not enough to more than enough”), existential or spiritual themes (e.g., “things that caused me great distress are perfectly ordained”), or impetus for action (“what to do in my life to course-correct”). The development of insight is key in many forms of psychotherapy (e.g., psychodynamic, cognitive-behavioral, mindfulness-based) and discussed also in emerging therapies based psychoactive plants [105]. Amazonian medicine’s concepts of ‘plants that teach’ (Teacher Plants, Master Plants) is relevant in this context [9,106]: in this (emic) view, the tobacco plant is understood as a conscious, agentic, and wise ‘other-than-human being’ (see Amazonian ontologies in this context [107,108] who, if ingested under the appropriate guidance, ritual, and dietary conditions, may offer humans teaching, often via dreams or visions [106,109]). Indeed, the patient reported instructive dreams (“I saw myself as a student […] hastily climbing stairs of knowledge, then was instructed to slow down and wait, since I am just a student, not a master […] I better understand my tendency to rush big things”), and described these dreams as unusual (e.g., “I find these dreams to be of a higher source”, “the dreams feel ordered, logical, clear”). Visions, on the other hand, were infrequent for the patient. Nonetheless, given the visionary states described in some Indigenous applications, several authors discussed tobacco as a hallucinogen, hypothesizing that MAO-inhibiting harmala (β-carboline) alkaloids present in the plant may be linked to such effects [13,82,110,111]. Indeed, while investigations of tobacco usually focus on nicotine, the role of other alkaloids of the plant for psychoactive effects has not yet been established [112]. β-carboline alkaloids are known to be important in the psychopharmacology of ayahuasca, via their interaction with DMT and independently (neuro-enhancing and antidepressant effects in pre-clinical studies) [113,114,115,116,117,118]. Nicotine is known to impact attentional processes [119,120], which could be linked to the reported clarity of mind and improvement of ADHD symptoms post treatment. However, other aspects (e.g., altered affective states, psychological insights, lifting of depressed mood) will require a more multifaceted explanatory model, possibly including other tobacco alkaloids, as well as extra-pharmacological factors inherent in this treatment. Indeed, the traditional interventions of the *Tabaquero* during the treatment and the *dieta* frame may co-determine outcomes and processes [76,88]. From the Amazonian emic view, aside from the plant’s direct biochemical effects, the tobacco plant as the *Tabaquero*’s principal Teacher Plant also participates in the healing process via its imparting of knowledge to the healer. The healer’s work (diagnosing, selecting/preparing medicines, treating patients) is thereby constantly informed by the Teacher Plant, via a carefully cultivated relationship involving different forms of interspecies communication [94,106,121,122]. Another extra-pharmacological aspect of the treatment may involve the relationship between healer and patient; the importance of the therapeutic alliance between therapist and patient is well-established in psychotherapy research (see Common Factors Theory) [123] and may well extend to traditional healing. The concepts of set and setting [124] may also have merit in this context, but, as we have argued elsewhere [88,125], are insufficient to account for the complexity of Indigenous applications of psychoactives. As an example, emesis in conjunction with tobacco, ayahuasca, and other plants is considered a key therapeutic mechanism in Amazonian medicine, associated with multiple kinds of depuration, depending on the specific plant at hand [76,90,91]. Related views around emesis and emetic plants and applications to treat nervous system conditions can also be found in other traditional medicines (e.g., Ayurveda, Thai, Zulu, or Xhosa [126,127,128]).

A limitation of this study was that we could not provide sample analyses of alkaloid content and dosage of the remedy. A dose-dependent effect can be inferred from the patient’s descriptions, with the small doses on days 5 and 6 followed by less intense psychological and somatic effects than on the other days. Furthermore, while a case report offers an important descriptive account for exploring a scientifically unknown treatment, its results may not be generalized, hence an additional limitation of the current work. Studies based on patient cohorts treated with this Amazonian therapy will be needed to assess effectiveness and basic pharmacokinetic/-dynamic parameters. Moreover, although the current study’s data was mainly based on patient self-reports (self-reported EMA and quantitative self-report scales), the qualitative interviews at pre- and post-treatment were conducted by a health professional who was part of the research team, which may be a potential source of bias. Finally, the current work assessed only short-term outcomes due to logistic limitations (the study coincided with the COVID-19 pandemic and was on hold for an extended period of time, which made further follow-up points impossible within the project period) forthcoming cohort studies should include mid- and long-term follow-up assessments to determine whether beneficial outcomes persist over time.

Nonetheless, the current work presents an important first step for the study of tobacco’s therapeutic possibilities and could serve as template for a larger study into therapeutic mechanism involved. It illustrates the traditional use of tobacco in healing ritual and offers for the first time a detailed descriptive account of how an Amazonian treatment involving drinking tobacco is experienced by a patient. This is significant in view of the globally prevalent harmful uses of tobacco [3,129,130], as it highlights a constructive usage of this plant as per Indigenous knowledge. It could in the long term open new accessible avenues in mental health (see worldwide gap in mental health services [34,35,36,37,38,39]), as well as economic opportunities for Indigenous populations with expertise in this context. Indeed, the transdisciplinary collaboration embedded in our research design is a strength of the current work, as it promotes inclusive research via knowledge co-production and benefit sharing [77,131]. Finally, this work is significant in the context of the aforementioned ‘psychedelic renaissance’. It suggests that tobacco, like psilocybin, MDMA, or LSD—all currently studied as promising clinical tools but previously seen as inevitably toxic [47,48,132,133,134,135,136]—should be further investigated for therapeutic potentials. Indeed, unlike MDMA or LSD, tobacco possesses a millennia-old history of therapeutic use in Indigenous American cultures [7,19,83,137,138,139]; a fresh look at the tobacco plant in close collaboration with traditional healers knowledgeable in its medical application is thus warranted.

## 4. Methods

The study was approved by the responsible ethics committees in Peru (PRISMA Comité Institucional de Ética en Investigación, CE0724.20) and Switzerland (University of Fribourg, 88-A1).

### 4.1. Design and Procedure

This work used a transdisciplinary case study design with a multi-method approach. We used validated psychological scales to assess symptoms before and after the tobacco-based treatment (quantitative), as well as ecological momentary assessment (EMA) [102] (also: experience-sampling, ambulatory assessment [103]) to document the patient’s process during the treatment (qualitative). EMA, which involves the repeated assessment of the variables of interest in the natural environment in which they occur, is increasingly used in psychology research due to the method’s capacity to capture experience in real- or near-real-time; contrary to the standard retrospective ratings (e.g., ‘How anxious did you feel over the last week?’), which are often biased by memory and heuristics [140,141,142]. More specifically, we here report an EMA using qualitative items (open questions), also labeled descriptive experience-sampling [143] or diary method [144,145,146], since we were particularly interested in the nature of experience (phenomenology) during the Amazonian tobacco-based treatment, in the experiencer’s own words and in relation to contextual factors [102,147]. The sampling schedule we used was interval-based with four measurement points per full treatment day. The qualitative items were administered twice per day; we concomitantly used quantitative EMA (single items rated from 0 to 6 to assess the momentary intensity of affective, cognitive, and somatic states) four times per day, but which will be reported in a separate paper.

The patient was thoroughly informed about the study and wrote an informed consent before the treatment was initiated. T.P. was then interviewed by a health professional (first author) and filled in the diagnostic self-report scales (see Section 4.2). She was handed a paper-and-pencil questionnaire folder for each treatment day, which she was instructed to fill in over the course of the day, according to the following schedule: 1st set of questions in the early morning upon getting up and up to 30 min later, 2nd set of questions in the late morning at 10:30/11 a.m. (before drinking the tobacco), 3rd set of questions in the afternoon at 3/4 p.m. (after drinking the tobacco, once intensity of effects has lessened), and 4th set of questions in the evening at 7/8 p.m. (before going to bed). One day after treatment completion, the patient was again interviewed by the health professional and again filled in the diagnostic self-report scales.

### 4.2. Measures

Standard, psychometrically validated psychological self-report scales were used for assessing pre- and post-treatment symptomatology: The *Symptom Assessment-45* (SA-45) [97,98] assesses mental health symptoms in nine domains, namely depression, anxiety, obsessive–compulsive, psychoticism, phobic anxiety, interpersonal sensitivity, paranoid ideation, somatization, and hostility; items are rated from 0 (*not at all*) to 4 (*extremely*). The *Perceived Stress Scale-10* (PSS-10) [148,149] assesses general psychological stress, with items rated from 0 (*never*) to 4 (*very often*). The *Patient Health Questionnaire-15* (PHQ-15) [100] assesses somatic symptoms, with items rated from 0 (*not bothered*) to 2 (*bothered a lot*). Finally, we used the *Positive* and *Negative Affect Schedule* (PANAS) [101] to asses affective states on two scales (positive and negative affect, 10 items each) rated from 1 (*very slightly or not at all*) to 5 (*extremely*). The EMA schedule was constructed from qualitative items of relevance based on general medical/psychological considerations, our previous research, and ethnographic literature on Amazonian tobacco [7,76]. It included questions in the mornings (quality and quantity of sleep, dreams in the preceding night), evenings (appetite, digestion, micturition, breathing, body temperature; how the visit of the healer was experienced; immediate effects after drinking the tobacco), as well as questions that were repeated in the mornings and evenings (experiences of healing, insights, instruction, transcendence experience).

### 4.3. Data Analysis

Data were analyzed using standard software (Microsoft Office and SPSS Statistics for Windows, Version 27.0, IBM Corp). For the quantitative questionnaires, scores were calculated by summing the respective items: nine domain scores (range 0–20 per domain) and one global severity index of the SA-45 [97,98], an overall psychological stress score (range 0–40) of the PSS-10 [149]; overall somatic severity (range 0–30) of the PHQ-15 [100]; positive affect and negative affect scores (range 10–50 each) of the PANAS [101]. Descriptive statistics and visual analysis were then used to explore differences in symptomatology before vs. after the treatment. For the qualitative items, we used the summarizing qualitative content analytic approach [150]: the main EMA content segments were defined as the main themes and used as main headings for a chronologically organized table. The items themselves served as subheadings (subthemes). The patient’s responses per item and day were then allocated to the corresponding subheading per day. If necessary, responses were slightly shortened, but the original wording was usually preserved in order to keep the meaning unaltered. If a description was repeated or spread out under several headings within the same assessment time point, the corresponding text was grouped together in order to improve the readability of the table.

## Figures and Tables

**Table 1:** Daily visits of the healer (*Tabaquero*) to patient.

Visit 1	Visit 2	Visit 3	Visit 4	Visit 5	Visit 6	Visit 7:	Corte (Day 8)
The healer explains the procedure; to drink water after the remedy, to then shower; that it may feel strong at first, but later relaxed. He explains that there will be many thoughts arising, some good ones, others bad, and recommends not to engage with negative thoughts so they can simply leave. The healer serves the remedy using traditional ritual, which includes icaros * (Amazonian chants) and blowing of tobacco (soplar). The p. drinks the remedy, drinks water, and a few minutes later vomits. Thereafter the healer leaves.	Brief conversation, then he serves the p. the tobacco remedy. She drinks water and vomits relatively soon. The healer departs.	The p. describes her feelings in relation to aspects of her life, including her illness. The healer listens closely and offers a few reflections. He serves her the tobacco, waits for her to vomit, and then leaves.	Brief conversation. The healer serves the remedy and waits. The p. exhibits unease as vomiting does not ensue promptly and effects seem to intensify. The healer instructs the p. to drink water and focus on her breathing. After a while she vomits. The healer tells her to shower and thereafter rest. He leaves.	Brief conversation. The healer tells the p. that yesterday there had been good work. He serves a very small quantity (very small sip) for her to drink, and then pours some of the liquid into her hand to take via the nostrils ^#^. The p. vomits and then the healer leaves.	The healer explains the p. that the day before when taking the tobacco via the nostrils, she had expelled a lot of phlegm, which was very good, since that phlegm had impacted her mental state. He gives her a small dose of the tobacco remedy to drink and tells her to try to keep the liquid in today without vomiting.	The healer serves the p. an inter-mediate dose, waits for her to vomit, and leaves.	The healer performs a brief prayer and ritual to close the dietary retreat. The patient is served an abundant meal and is recommended to rest thereafter.

Note: P. = patient. * For the concepts of *icaros* and *soplar* see [59,92,93,94]. ^#^ A form of tobacco application called ‘*singar*’ in Peruvian traditional medicine [95,96].

**Table 2:** Psychological and somatic symptoms at pre- and post-treatment assessment.

	Pre-Treatment	Post-Treatment
SA-45		
Global Severity Index	47	2
- Anxiety	5	0
- Depression	11	0
- Obsessive/Compulsive	7	1
- Phobic Anxiety	1	1
- Hostility	5	0
- Interpersonal Sensitivity	9	0
- Paranoid Ideation	8	0
- Somatization	1	0
- Psychoticism	0	0
PHQ-15		
Overall somatic symptoms	8	0
- Stomach pain	0	0
- Back pain	2	0
- Pain in arms, legs, or joints (knees, hips, etc.)	1	0
- Menstrual cramps, problems with period	0	0
- Headaches	0	0
- Chest pain	0	0
- Dizziness	1	0
- Fainting spells	0	0
- Feeling heart pound or race	1	0
- Shortness of breath	0	0
- Constipation, loose bowels, or diarrhea	0	0
- Nausea, gas, or indigestion	0	0
- Feeling tired or having low energy	2	0
- Trouble sleeping	1	0
PSS-10		
Overall psychological stress	23	0
PANAS - Positive affect	18	42
- Negative affect	22	10

Note: SA-45 = Symptom Assessment-45. PHQ-15 = Patient Health Questionnaire-15 (minimal severity: score of 0–4, low severity: score of 5–9; medium severity: score of 10–14, high severity: score of 15–30 [100]. PSS-10 = Perceived Stress Scale; PANAS = Positive And Negative Affect Schedule. Higher scores indicate higher severity of symptoms, except for PANAS positive affect.

**Table 3:** Ecological momentary assessment (experience-sampling) during the treatment week.

		Day 1	Day 2	Day 3	Day 4	Day 5	Day 6	Day 7	Corte (Day 8)
	**This morning got up at…**	-	6:00	6:00	5:45	5:30	7:00	05:45	06:00
**Morning: Experiences of the Preceding Night**	**Yesterday went to bed at…**	-	20:45	20:30	20:45	20:30	20:00	20:00	21:00
	**Fell asleep after…**	-	a long time	a long time	a bit	a bit	a long time	a bit	a bit
	**Awoke during the night at…**	-	3:30	3:30	3:30	2–3	00–3	00:00	00:00
	**Sleep quality (0-very bad, 6-very good)**	-	2	4	4	5	3	5	4
	**Dreams, Instructions, important insights, healing experiences during night**	-	I can’t remember the dreams well; many animals were searching for me; a horse, a reptile.	- Dreams with general life instructions through storytelling: I saw myself as a student again, maybe 11–12, with a backpack at school. I was hastily climbing stairs of knowledge, then was instructed to slow down and wait, since I am just a student, not a master. - Guidance from higher powers, dreams from spirit. - I better understand my tendency to rush big things. - I awoke with painful thoughts and observed them as they flew away…	I remember dreaming but can’t recall them.	- The dreams were like a tapping on the shoulder and a whisper–information to support my healing work. They were reminders or reinforcements of some of the day’s learning. - To have gratitude for every moment–to be here now is extremely lucky, to have means and knowledge to access this healing very unusual. - An insight about my abundance: switch the perspective from having not enough to more than enough.	- I was dreaming that the wrong alignment at work is self-injurious…like putting a spell on myself and being in emotional or psychological danger. - New insights for my healing. - Themes of self-protection especially related to work.	Dreams with themes of self-care and putting my needs first; to take joy in providing for myself and my needs– as if I am giving myself Christmas gifts.	- Guidance for how to handle personal and business aspirations; I was instructed to hold the cards close to the chest– to be very selective about who I share my ideas with, instead of seeking encouragement and excitement. That the insights are for me to grow and develop alone until the right time. - The dreams feel ordered, logical, clear, and supportive to the healing journey - I find these dreams to be of a higher source, divine.
**Healer’s Visit**	**Did the visit feel supportive/help understand something?** *(See Table 1 for observer descriptions of the visits)*	Yes, he has such a gentle, nurturing energy, I felt glad to be in his presence. It helped me understand the numerous ways in which I’m limiting myself, problematic patterns. Clear and new ways of being emerged.	Yes, his words were sparse but brought me to tears. I felt seen. I understand surrendering to my path more fully, with less tension.	Yes	Yes, he can peer into the soul. Made me reflect on the process of spiritual awakening—how to seek God instead of the self.	Yes	Yes supportive, understandings still unclear	Yes	
**Immediate Effects after Drinking Tobacco**	**Unusual body sensations**	-	-	Heat	Electricity surging through my whole body. Agonizing nausea, heat, dizziness.	Tingling, nausea.	Imperceptible after drinking a spoonful or so. Mild effects within 1–2 h.	Stomach churning.	
	**Vomiting**	3 bouts	3 episodes	2	2	1	No	2	
	**Changes in attention or thought processes**	Yes. My thoughts were remarkably clear and complete. Complex progressions of insight with grounded and sustained attention. I noticed how novel this felt because I have ADHD and struggle with sustaining attention and thought, especially complex problem solving.	Yes. Attuned, alert, clear-headed.	Yes. I am so still and clear. Solutions and understandings are completely available, no uncertainty.	Yes. My mind again cleared. This time it gave me a break from all the processing. I had no thoughts to tease through, just mental openness. I spent my day creating art for the first time in many years.	No. Very small dose today, effects were gentle.	Yes. Calm quiet thinking.	Yes. Calm and direct thoughts, almost instructional, peaking around bedtime.	
	**Changes in emotions**	Yes, immediately emotional, in intense waves; relief, gratitude, grief, joy came zipping out of me. I knew the purge would relieve me of sufferings. I thought and felt the sense of ‘Finally’; I was so grounded and clear-headed. Released shame and grief that I have been repeatedly punishing myself with.	Yes. Calm, assured, certain, complete.	Yes. I have written 10 pages of deeply emotional insights. I feel whole.	Yes. I was fearful before. The medicine took it away; even the fear of dying.	-	Yes	Yes. A great flattening of intense emotions, either depressive or manic—just grounded peace.	
	**Visual effects**	Sort of. I was slipping into an in-between half dream state and felt like I was being given a story or receiving storytelling that was spiritually important, but I can’t remember what it was.	-	-	Yes. A moving set of circles with beams shooting at a cross; a snake skeleton.	-	-	-	
**Before Going to Sleep: Experiences from the Afternoon/Evening**	**Insights**	About relationships, work, spirituality, shamanic healing, ways of being, connection. I had directions repeatedly and took many notes. Things to investigate further. Ideas outside of myself.	About how to love my family and receive love. How presence is the key to joy.	About my patterns—all over. Family, work, how I exist with the world, my self-limiting beliefs.	About my difficulty with surrender and trust. My fear of dying. Spent all day reflecting on trusting the medicine and having patience as it works on me.	A focus on my control tendencies, impatience, as if the medicine worked differently by taking a day off.	That my highest and greatest good is only available if I heal my spirit and mind first.	About what to do in my life to course-correct, like an actual laundry list. So straightforward and clear, and in order of priority.	
	**Healing experience**	Many things feel spontaneously clear…maybe resolved.	My nervous system and mind feel perfectly still. The healing felt very grounded and physical.	Full body presence, peace, calm.	My patience and self-compassion are growing.	I do believe healing was happening, but without feeling the tobacco much, it was a different type.	I am stronger daily; I do not feel sick.	I acknowledged my tendency to run, to seek novelty, to fluctuate across highs and lows, to overexert control. Honestly the first time I have had real insight about this.	
	**Experience of being taught something**	I journaled 3 intense pages of instructions to and for myself. I learned some gospel.	Action through nonaction is the path to my highest good.	Innumerable—one after another. Clearly realized how I am mirror with others—what frustrates me with them are my unhealed spots.	I was taught about my eagerness to please and be validated by others. Noticing so many subtleties of my behavior.	-	I felt a deep “settling in”, a fuller awe.	What developed over the day became a clear personal philosophy, I wrote it down like my life’s work, connecting dots. It seemed like I was getting the building blocks for my business (future) or eventual book chapters.	
	**Spiritual/energy-related/transcendence experiences**	I am too unfamiliar with this medicine to try to explain yet. Energetic but not completely ethereal like ayahuasca. Grounded energy.	I see the last six months crystal clear—the things that caused me great distress are perfectly ordained.	- So profound. The medicine feels like it is opening me. While navigating to the toilet with a flashlight to purge, I realized there is no better use of my time than this. - God work.	100,000 volts of electricity; like an energetic near-death experience.	-	Not rocket-ship spiritual…but a continued awakening.	Insight coming one after another, after dark, I could hardly put my pen down.	
**Changes in Physiological Functions Today**	**Appetite**	Diminished, bites of food only	Diminished	Even less	Normal	Normal	Diminished	Normal	
	**Body temperature**	Adequately warm	Adequately warm	Adequately warm	Adequately warm	Adequately warm	Adequately warm	Adequately warm	
	**Breathing**	Normal	Slower and calmer	Normal	Normal, only for 5 min after the medicine rapid	Normal	Normal	Normal	
	**Micturition**	Normal	Normal	Normal	Normal	Normal	Normal	Normal	
	**Digestion**	Diarrhea, felt like part of the purging	Diarrhea	Slight diarrhea	Normal	Diarrhea	Normal	Normal	

Note: since the treatment started and ended around noon on the first and last day, some fields are blank.

## Data Availability

The data are not publicly available to protect the privacy of the patient.
